# A Robust Nonrigid Point Set Registration Method Based on Collaborative Correspondences

**DOI:** 10.3390/s20113248

**Published:** 2020-06-07

**Authors:** Xiang-Wei Feng, Da-Zheng Feng

**Affiliations:** National Laboratory of Radar Signal Processing, Xidian University, Xi’an 710071, China; fengxiangwei@stu.xidian.edu.cn

**Keywords:** nonrigid point set registration, structural feature, absolute distance, relative distance, correspondence

## Abstract

The nonrigid point set registration is one of the bottlenecks and has the wide applications in computer vision, pattern recognition, image fusion, video processing, and so on. In a nonrigid point set registration problem, finding the point-to-point correspondences is challengeable because of the various image degradations. In this paper, a robust method is proposed to accurately determine the correspondences by fusing the two complementary structural features, including the spatial location of a point and the local structure around it. The former is used to define the absolute distance (AD), and the latter is exploited to define the relative distance (RD). The AD-correspondences and the RD-correspondences can be established based on AD and RD, respectively. The neighboring corresponding consistency is employed to assign the confidence for each RD-correspondence. The proposed heuristic method combines the AD-correspondences and the RD-correspondences to determine the corresponding relationship between two point sets, which can significantly improve the corresponding accuracy. Subsequently, the thin plate spline (TPS) is employed as the transformation function. At each step, the closed-form solutions of the affine and nonaffine parts of TPS can be independently and robustly solved. It facilitates to analyze and control the registration process. Experimental results demonstrate that our method can achieve better performance than several existing state-of-the-art methods.

## 1. Introduction

Nonrigid point set registration is broadly applied in computer vision fields, such as face recognition, fingerprint matching, object tracking, remote sensing, medical image processing, and simultaneous localization and mapping (SLAM) [1,2,3,4,5,6].

The feature points that are used in the point set registration methods are extracted from the corresponding images, which may include edges [7], corners [8], SIFT [9], ORB [10], and so on. These feature point sets can well preserve the crucial structural features of images. Let the model point set and the scene point set be represented by $X_{M\times D}={\left[x_{1}^{T},x_{2}^{T},\ldots ,x_{M}^{T}\right]}^{T}$ and $Y_{N\times D}={\left[y_{1}^{T},y_{2}^{T},\ldots ,y_{N}^{T}\right]}^{T}$, respectively, where M and N are the number of points in the point sets and D is the dimension of the feature points. The aim of the point set registration methods is to find the interpolation function f(X;θ) to recover the spatial transformation from X to Y, where θ represents the parameters of the interpolation function. Once the correspondences are determined, a set of equations can be established to solve the transformation functions. However, the correspondences are unknown in practice. Point set registration can be divided into the two closely related subproblems: determine the correspondences and estimate the transformation. Nonrigid transformation is flexible and irregular so that the complicated interpolation functions with a large number of parameters have to be adopted. Thus, recovery of the nonrigid transformation function tends to be an ill-posed problem unless there are a sufficient number of correct correspondences. However, the image degradations, including large deformations, noise, outliers, occlusion, and rotation, make it hard to find adequate correct correspondences. For instance, many objects are composed of several parts following a certain distribution pattern, such as Chinese characters. The image degradation can lead to that several parts of the object significantly deviate from their original positions. In this case, the Euclidean distance between the model points from these parts and the scene points from the corresponding parts will be large. If the methods merely rely on the Euclidean distance to determine the correspondence, it will be difficult to establish the accurate correspondences among the points from deviating parts. However, due to physical and geometric constraints in the real world, the local structures are stable and reliable even though the point set is going through severe degradations. For example, whether one is smiling or laughing, the points that represent the five sense organs on the face can well maintain their own internal structural relations. The local descriptors, such as shape context (SC) [11], can be adopted to generate the descriptions of the local structures around the points. We can retrieve the pointwise correspondences by comparing the similarities of the local structural descriptions rather than Euclidean distance between their spatial locations. It can help to preserve the relations of the point pairs in which the spatial locations are far, but the local structures are similar. Therefore, the local structural descriptions-based correspondences can be regarded as a good supplement.

In this paper, we propose a robust method for the nonrigid point set registration by fusing different structural features to determine the correspondences between the point sets to be matched. The structural features are classified into two types. The first type is the spatial location of a point. Ideally, if the spatial transformation between two point sets is completely recovered, the feature points should approximately overlap with their corresponding points. We can directly compare the spatial locations of two points to measure their corresponding relations, and the distance between the spatial locations of two points is defined as the absolute distance (AD). Many kinds of distance can be employed to define the AD, such as Mahalanobis distance, Manhattan distance, and Euclidean distance. Because Euclidean distance is simple, general, and convenient to calculate, we adopt Euclidean distance as the AD. Gaussian kernel is employed to define the AD-correspondences based on the AD. It is convenient to directly convert AD into AD-correspondence using Gaussian kernel. Further, Gaussian kernel can assign strong and weak correspondences to the point pairs with small and large AD, respectively. Moreover, through adjusting the variance of Gaussian kernel, we can control the search range of the AD-correspondences. This can be utilized to determine the corresponding relations from coarse to fine. The second type is the distributions of the remaining points around the selected point. We use the local descriptors, such as SC, to measure the relative distance (RD) of the local structures between the points and determine the RD-correspondences. SC can efficiently inform the context structural information that relies purely on the points coordinates. It is translation-invariant and can maintain stable performance under the degradations of the local deformations, noise, and many outliers [11,12]. Besides, it can also be invariant to rotation by adopting the tangent vector at each point as the positive X-axis. By introducing the neighborhood corresponding consistency, the adverse effects derived from mismatches of RD-correspondences can be suppressed. According to the proposed heuristic method, AD-correspondences and RD-correspondences are combined to establish the collaborative correspondences. Based on the obtained correspondences, recovery of the transformation function can be treated as a least square problem. TPS is adopted as the transformation function. Its parameters can be explicitly divided into affine and nonaffine parts, and thus TPS possesses the clear physical meaning [13]. In our method, the affine and nonaffine parts are theoretically separated and can be independently solved. It makes the registration process have the clear physical meaning, and provides convenience to analyze and control the registration process. Experimental results demonstrate that our method outperforms several existing state-of-the-art methods in most scenarios.

The remainder of this paper is organized as follows: Section 2 reviews the previous work, Section 3 presents the proposed method, Section 4 gives convergence analysis, Section 5 discusses the computational complexity, Section 6 gives the experimental results, and finally, Section 7 concludes this paper.

## 2. Previous Work

In recent decades, a number of good methods have been developed to deal with the nonrigid point set registration problems. Here, we give a brief review.

The method of iterative closest points (ICP) [14,15] may be the most popular iterative point registration method. This technique establishes a binary matrix to represent the corresponding relationship based on the nearest neighborhood strategy. The ICP method requires that the two point sets are close enough, otherwise, a number of false correspondences are generated to severely affect the performance. In order to improve the robustness of ICP, many famous methods are developed by relaxing the binary corresponding constraints. Chui et al. [16,17] proposed the famous thin plate spline-robust point matching (TPS-RPM) method. In this work, the authors designed a general framework to iteratively determine the fuzzy correspondences and estimate the spatial transformation based on soft assignment and deterministic annealing. Another representative work is coherent point drift (CPD) that models the point set registration as a probability density estimation problem [18,19]. This method lets one point set be taken as the centroids of Gaussian mixture model (GMM), and achieves the registration by making the GMM centroids to fit the other point set under the expectation–maximization (EM) framework. Besides, in [20], the point sets are treated as two kernel densities, and the point set registration is achieved by maximizing the kernel correlation (KC) between them. An improved version of KC work, GMMREG, can be found in [21] by taking the L2 distance to measure the similarity between two Gaussian mixtures. Later, Ma et al. [22] and Hasanbelliu et al. [23] refined the measure of the similarity between Gaussian mixtures by L2E (namely, RPM-L2E) and Cauchy–Schwarz divergence. By refining the models to capture the spatial distributions of point sets, Tao et al. [24], Wang et al. [25], and Zhou et al. [26] accomplished the point set registration using nonuniform Gaussian mixture models, asymmetric Gaussian mixture models, and Student’s *t* mixture models, respectively. The above methods [14,15,16,17,18,19,20,21,22,23,24,25,26] mainly utilize the spatial locations of the feature points. However, they neglect the local structures around the feature points that are very important to help determine the corresponding relationship between different point sets. 

Through introducing the local geometric characteristic, many good methods were developed [27,28,29,30,31,32,33,34,35,36,37]. Zheng et al. [27] proposed a robust nonrigid matching method by preserving local neighborhood structures (RPM-LNS). The local structures were interpreted as a simple graph, and were preserved by maximizing the number of matched edges between two corresponding graphs. Yang et al. [28] proposed GLMDTPS by designing a global and local mixture distance to determine the corresponding relationship. Ma et al. [29] developed a robust point method by preserving the global and local structures (PRGLS). In [30], the authors used k-connected neighbors to construct connectivity matrix, and cast the local structures preservation to minimize the weighted least square error. In [31], the authors proposed the mixture structure descriptor to define the pointwise distance, and designed two energy functions to simultaneously preserve the global and local structures, respectively. In [32,33], Ma proposed a novel method named “MR-RPM” by adopting the manifold regularization to catch the underlying structure of the point sets and help to learn the transformation. In [34], the authors achieved the nonrigid point registration by using two local descriptors of the connectivity matrix and Laplacian coordinate to preserve the geometry structures. In [35,36], Song and Fan proposed a nonrigid registration method via global–local topology preservation (GLTP). The local linear embedding (LLE) was employed to preserve the local topological structures. By taking a local geometric constraint as a regularizer, and designing a semisupervised EM framework, a feature-guided Gaussian mixture model for point set registration was presented in [37]. Next, a brief analysis of these methods is given in [27,28,29,30,31,32,33,34,35,36,37]. In conclusion, the first strategy of the methods is to estimate the correspondences by fusing various structural features, and the second is to introduce spatially constraints to preserve local structural topology. The above two strategies can be utilized together. These methods are very constructive and notable to improve utilization efficiency of the structural information. There are still some details that need to be addressed. For the first strategy, the methods in [27,29,30,31,32,33,37] employ multiplicative model to fuse the global and local structures. It mainly concentrates on the point pairs that have both small Euclidean distance and similar local structures. It is efficient when the point sets are compact and simple. However, this model might be not able to find enough correspondences when the point sets have complicated structures, such as Chinese characters. Besides, most of them take less account of efficient design to handle mismatches. The performance of these methods would be sensitive to the large deformations and outliers. For instance, the mixture distance in [28] can be used to search accurate correspondences without outliers. Nonetheless, the outliers can reduce the discriminative ability of the mixture distance, which leads to generate mismatches to degrade performance. For the second strategy, [27,30,31,32,33,34,35,36,37] adopt different spatially constraints, such as the neighboring connectivity matrix and classical manifold regularization techniques, to maintain stability of local structures in the registration process, which can be treated as a regularizer. This assumption is very reasonable. But, once the accurate corresponding relationship is not established, this strategy does not perform well. Besides, in our paper, we employ TPS as the spatial transformation. Its parameters can be explicitly decomposed into the affine and nonaffine parts. Therefore, we can respectively add regularization terms to the affine and nonaffine transformation. Experimental results show that it can efficiently prevent arbitrary spatial transformation. Thus, we focus on establishing accurate corresponding relationship in this paper.

In order to handle large deformations, Du et al. [38] developed a novel method based on heuristic tree. This method first built the heuristic tree using the shape similarities that are derived by affine ICP. Then TPS and CPD can possessively accomplish the nonrigid point set registration along the tree. This method requires a set of point sets to build the tree. In [39], the local structure preservation theories were exploited to remove mismatches for improving the corresponding accuracy, which is named “LPM.” This method requires preregistration to determine putative one-to-one correspondences. Graph techniques were also exploited for point set registration problems [40,41,42,43]. In [40], the graph centralities that are combined with the spatial information of point sets were regarded as priors to be embed with CPD. In [41,42], the authors developed a graph-based point registration method (namely, FGM). They factorized the large pairwise affinity matrix into smaller matrices that encode the local structure of each graph and the pairwise affinity between edges. In [43], a new third-order graph matching technique was developed to determine the correspondences. Graph-based methods provide a novel and creative way to handle with the nonrigid point set registration problems. However, graph-based methods should further improve their performance when there exist data degradations of noise and outliers. 

There are also many other excellent methods for nonrigid point registration and we just cite a few here. More methods can be found in good reviews like [44,45].

## 3. Methods

In this section, we first defined the AD and AD-correspondences and the RD and RD-correspondences. Then the AD-correspondences and RD-correspondences are combined by the proposed heuristic method to determine the corresponding relationship between two point sets. Once the correspondences are obtained, the transformation estimation can be modeled as a least square problem. Fortunately, we can independently get the closed-form solutions of the affine and nonaffine parts of TPS, when the correspondences are given. Subsequently, we introduced the deterministic annealing scheme and analyzed the convergence properties. Finally, we gave the computational cost of the proposed method.

### 3.1. Absolute Distance and Correspondence

AD is defined as the Euclidean distance between the spatial locations of the points. Given a point $f\left(x_{m}\right)$ from the model point set f(X) and a point $y_{n}$ from the scene point set Y as the reference points, the AD between $f\left(x_{m}\right)$ and $y_{n}$ is denoted as

(1) $$d_{AD}\left(f\left(x_{m}\right),y_{n}\right)={\left\|f\left(x_{m}\right)-y_{n}\right\|}^{2}.$$

Based on AD, several available forms of correspondences can be established. The ICP approach is broadly used in practice. It employs AD and the nearest strategy to define pointwise correspondences. The correspondence between $f\left(x_{m}\right)$ and $y_{n}$ can be expressed as

(2) $$c_{ICP}\left(f\left(x_{m}\right),y_{n}\right)=\left\{ \begin{array}{l} 1\;d_{AD}\left(f\left(x_{m}\right),y_{n}\right)\leq \min\left\{d_{\min}^{AD},\kappa \right\}\\ 0\;others \end{array}\right.,$$

where $d_{\min}^{AD}=\min\left\{d_{AD}\left(f\left(x_{m}\right),y_{1}\right),\ldots ,d_{AD}\left(f\left(x_{m}\right),y_{N}\right)\right\}$ and κ is a threshold to reject the correspondences with large AD. However, ICP only utilizes the structural information between the reference point and its nearest point in the other point set. It is too rough to detect the complicated relations between point sets. If the initial positions of the point sets are not overlapping enough, a number of false correspondences are generated in the ICP approach. In addition, because the nonrigid deformations vary greatly in different parts of the same object, it is difficult to set an appropriate threshold to reject the putative correspondences. 

In our paper, we use Gaussian kernel to define the AD-correspondence between $f\left(x_{m}\right)$ and $y_{n}$, which is written as

(3) $$c_{AD}\left(f\left(x_{m}\right),y_{n}\right)=\exp\left(-d_{AD}\left(f\left(x_{m}\right),y_{n}\right)/T\right),$$

where T is the variance or temperature of the Gaussian kernel. According to Equation (3), the corresponding relationship between $f\left(x_{m}\right)$ and scene point set Y can be represented by

(4) $$c_{AD}\left(f\left(x_{m}\right),Y\right)=\sum_{n=1}^{N}c_{AD}\left(f\left(x_{m}\right),y_{n}\right).$$

At the beginning of the registration process, because the AD between the model point X and the scene point Y is usually large, it is difficult to establish the accurate pointwise correspondences. A robust way is to utilize a Gaussian kernel with high temperature T to preserve the sufficient correspondences as the candidates. Notably, although the point pairs with small AD initially possess a closer relationship than the point pairs with large AD, the point pair with the smallest AD is not necessarily the correct correspondence. Different from ICP arbitrarily employing 0–1 correspondence, our method not only retains the point pairs with the smallest AD to participate in the determination of the correspondences but also leaves the chance for all the point pairs to improve their roles gradually. When the temperature T decreases, Gaussian kernel pays more attention on the local structures, and the AD-correspondences are increasingly refined. The correspondences of point pairs with small AD play increasingly important roles. Specifically, when the temperature T is low enough, this approach corresponds to the nearest strategy.

### 3.2. Relative Distance and Correspondence

As the concise representations of the images, the point sets preserve abundant local structures. The local structures are stable and reliable, despite the point set going through severe degradations because of the physical and geometric constraints in the real world. Given the reference points $f\left(x_{m}\right)$ and $y_{n}$, we can detect their correspondences by comparing the similarity of their surrounding local structures, where the similarity is defined as RD.

Herein, SC is employed as the local structural descriptors. SC adopts a group of well-designed bins in log-polar space to catch the spatial distributions of the remaining points with respect to the reference points. Suppose $h_{m}^{X}\left(s\right)$ and $h_{n}^{Y}\left(s\right)$ are the number of points in s−th bin with respect to the reference points $f\left(x_{m}\right)$ and $y_{n}$, respectively. The RD between two points is defined by the $\chi^{2}$ test statistic, which is denoted as

(5) $$d_{RD}\left(f\left(x_{m}\right),y_{n}\right)=\frac{1}{2}\sum_{s=1}^{S}\frac{{\left[h_{m}^{X}\left(s\right)-h_{n}^{Y}\left(s\right)\right]}^{2}}{h_{m}^{X}\left(s\right)+h_{n}^{Y}\left(s\right)}.$$

Based on RD, the preliminary RD-correspondences can be recovered as one-to-one mapping by bipartite graph matching techniques, such as the Hungarian method [46]. If $f\left(x_{m}\right)$ is matched to $y_{n}$, the RD-correspondence between two points is TRUE and ${\tilde{c}}_{RD}\left(f\left(x_{m}\right),y_{n}\right)=1$. Otherwise, the RD-correspondence between two points is FALSE and ${\tilde{c}}_{RD}\left(f\left(x_{m}\right),y_{n}\right)=0$.

However, it cannot be guaranteed that there is no mismatch among the preliminary RD-correspondences. Herein, we exploit neighboring corresponding consistency to assign confidence for each correspondence to restrain the adverse impacts of mismatches. The main motivation is that the neighborhoods of points that are correctly matched should also be matched. Suppose the corresponding point of the model point $f\left(x_{m}\right)$ is $y_{n}$. First, we find K closest points of $f\left(x_{m}\right)$ in model point sets, and K closest points of $y_{n}$ in scene point sets, when the RD-correspondences are TRUE. The closest points of $f\left(x_{m}\right)$ and $y_{n}$ are, respectively, denoted as $\left\{f\left(x_{\pi \left(m,k\right)}\right)\right\}$ and $\left\{y_{v\left(n,k\right)}\right\}$, where k=1,2,…,K, π(m,k) and v(n,k) represent the serial number of the k−th closest point to $f\left(x_{m}\right)$ and $y_{n}$, respectively. Second, based on the one-to-one correspondences determined by the SC, we can obtain the corresponding points of $\left\{f\left(x_{\pi \left(m,k\right)}\right)\right\}$ in the scene point sets, which are denoted as $\left\{y_{\bar{v}\left(n,k\right)}\right\}$, where k=1,2,…,K and $y_{\bar{v}\left(n,k\right)}$ is the corresponding point of $f\left(x_{\pi \left(m,k\right)}\right)$. Third, we use consistent distance $d_{C}$ to evaluate the consistency between $\left\{y_{v\left(n,k\right)}\right\}$ and $\left\{y_{\bar{v}\left(n,k\right)}\right\}$

(6) $$d_{C}\left(f\left(x_{m}\right),y_{n}\right)=\sum_{k=1}^{K}{\left(\left\|y_{v\left(n,k\right)}\right\|-\left\|y_{\bar{v}\left(n,k\right)}\right\|\right)}^{2}.$$

Subsequently, the confidence between $f\left(x_{m}\right)$ and $y_{n}$ is defined as follows

(7) $$\eta_{mn}=\exp\left[-d_{C}\left(f\left(x_{m}\right),y_{n}\right)/T\right].$$

The final RD-correspondences are determined as follows

(8) $$c_{RD}\left(f\left(x_{m}\right),y_{n}\right)=\eta_{mn}{\tilde{c}}_{RD}\left(f\left(x_{m}\right),y_{n}\right).$$

As illustrated by Equations (6)–(8), if $\left\{y_{v\left(n,k\right)}\right\}$ and $\left\{y_{\bar{v}\left(n,k\right)}\right\}$ are highly consistent, the consistent distance is small, and the corresponding confidence between $f\left(x_{\pi \left(m,k\right)}\right)$ and $y_{n}$ is high. Otherwise, the confidence is low. Specially, if $\left\{y_{v\left(n,k\right)}\right\}$ and $\left\{y_{\bar{v}\left(n,k\right)}\right\}$ are identical, the confidence $\eta_{mn}$ equals 1. Besides, the correspondences are also involved with the temperature T. We can maintain much RD-correspondences by employing high temperature T. Through reducing T, the requirement of the corresponding consistency becomes more and more rigorous. In the end, when T is low enough, only the RD-correspondences with zero or approximately zero consistent distance are preserved.

### 3.3. Correspondence Collaboration

As illustrated in Section 3.1 and Section 3.2, AD-correspondences and RD-correspondences are derived from different structural features. The point sets contain multiple structural features in scales and patterns. In order to efficiently utilize the structural features, the methods cannot merely rely on single type of structural feature. Here, we fuse various structural information by the collaborative correspondences as follows

(9) $$c\left(f\left(x_{m}\right),y_{n}\right)=T^{\rho }\cdot c_{RD}\left(f\left(x_{m}\right),y_{n}\right)+c_{AD}\left(f\left(x_{m}\right),y_{n}\right),$$

where ρ is a parameter to control the effects of the RD-correspondence. In the registration process, AD-correspondences and RD-correspondences are complementary and collaborative to handle various cases. Given reference points $f\left(x_{m}\right)$ and $y_{n}$, in one case when the AD is small, the AD-correspondence is strong and plays a fundamental role in the collaborative correspondence. Moreover, if the RD-correspondence between $f\left(x_{m}\right)$ and $y_{n}$ is TRUE with high confidence, the collaborative correspondence is further strengthened by the RD-correspondence. Thus, this pair of points is assigned a strong corresponding relationship and plays a dominant role in the following registration process. If the RD-correspondence is FALSE or TRUE with low confidence, the collaborative correspondence can still maintain strong relation because of the AD-correspondence. In another case, the nonrigid deformations frequently make some local areas move away from their original positions, which leads to the AD being large and the AD-correspondence being weak. However, by comparing the RD of the local structures, RD-correspondences of the points in these local areas are likely to be established. The collaborative correspondences of these points are mainly determined by RD-correspondences and play an auxiliary role in the following registration process. Notably, these corresponding local areas are matched more accurately step by step, which can lead to smaller ADs and stronger AD-correspondences. Thus, an increasing number of point pairs that simultaneously have strong collaborative correspondences can help us improve the registration accuracy.

### 3.4. Transformation Estimation

Based on the collaborative correspondences, the corresponding relationship and confidence between $f\left(x_{m}\right)$ and $y_{n}$ can be defined as

(10) $$p_{mn}=\frac{c\left(f\left(x_{m}\right),y_{n}\right)}{\varsigma \frac{N}{M}+\sum_{q=1}^{N}c\left(f\left(x_{m}\right),y_{q}\right)},$$

where ς is a parameter to suppress outliers, $P={\left\{p_{mn}\right\}}_{M\times N}$ is the corresponding matrix, and let $\sum_{n=1}^{N}p_{mn}=1$ by means of normalization. After the corresponding relationship is determined, the transformation estimation can be treated as a least square problem. We adopt the TPS function as the transformation function [13]. The greatest merit of TPS is that its parameters can be explicitly decomposed into the affine and nonaffine parts. Let ${\bar{x}}_{m}=\left[1,x_{m}\right]$ and ${\bar{y}}_{n}=\left[1,y_{n}\right]$ be the homogeneous coordinates of $x_{m}$ and $y_{n}$, respectively. The new location of point ${\bar{x}}_{m}$ after TPS transformation is represented as follows

(11) $$f\left({\bar{x}}_{m}\right)={\bar{x}}_{m}d+\phi_{m}w,$$

where $d\in {\mathbb{R}}^{(D+1)\times D}$ and $w\in {\mathbb{R}}^{N\times D}$ represent the affine and nonaffine parts, respectively, and $\phi_{m}$ is the m−th row of kernel matrix $\Phi =\left\{{\left\|{\bar{x}}_{s}-{\bar{x}}_{l}\right\|}^{2}\ln\left\|{\bar{x}}_{s}-{\bar{x}}_{l}\right\|\right\}\in {\mathbb{R}}^{M\times M}$. Let $\bar{X}={\left[{\bar{x}}_{1}^{T},{\bar{x}}_{2}^{T},\ldots ,{\bar{x}}_{M}^{T}\right]}^{T}$ and take QR decomposition of $\bar{X}$, the affine and nonaffine warping spaces are separated, which is written as

(12) $$\bar{X}=QR=\left[Q_{1}Q_{2}\right]R,$$

where $Q_{1}\in {\mathbb{R}}^{M\times \left(D+1\right)}$ and $Q_{2}\in {\mathbb{R}}^{M\times \left(M-D-1\right)}$ are orthogonal. Because the boundary constraint of TPS is given by ${\bar{X}}^{T}w=0$, we must have

(13) $$w=Q_{2}\tilde{w}.$$

Let $\bar{\Phi }=Q_{2}^{T}\Phi Q_{2}$ and $\tilde{\Phi }=Q_{1}^{T}\Phi Q_{1}$, then there must be $\Phi =Q_{1}\tilde{\Phi }Q_{1}^{T}+Q_{2}\bar{\Phi }Q_{2}^{T}$. With the above analysis and without changing transformation, Equation (11) can be rewritten into

(14) $$f\left({\bar{x}}_{m}\right)={\bar{x}}_{m}d+q_{2,m}\bar{\Phi }\tilde{w},$$

where $q_{2,m}$ is the row m of $Q_{2}$. In order to smooth the nonaffine parts of TPS, a regularization term $\lambda_{1}w^{T}\Phi w$ is added to the cost function. Here, $\lambda_{1}$ is a free parameter to control the effects of the regularization term. Based on the above analysis, the cost function can be written as

(15) $$\min J\left(d,\tilde{w}\right)=\sum_{m=1}^{M}{\left\|{\tilde{y}}_{m}-{\bar{x}}_{m}d-q_{2,m}\bar{\Phi }\tilde{w}\right\|}^{2}+\lambda_{1}T\mathrm{tr}\left({\tilde{w}}^{T}Q_{2}^{T}\Phi Q_{2}\tilde{w}\right)+\lambda_{2}T\mathrm{tr}\left[{\left(d-I\right)}^{T}\left(d-I\right)\right],$$

where ${\tilde{y}}_{m}=\sum_{n=1}^{N}p_{mn}{\bar{y}}_{n}$ can be treated as the newly estimated positions, ${\left(d-I\right)}^{T}\left(d-I\right)$ is a term to regularize the affine transformation, in which $\lambda_{2}$ is a free parameter to control it. Notably, $\lambda_{2}$ is much smaller than $\lambda_{1}$ in order to give affine transformation more freedom. If considering Equation (13), the compact form of Equation (15) can be represented as follows

(16) $$\min J\left(d,\tilde{w}\right)={\left\|\tilde{Y}-Q_{1}Rd-Q_{2}\bar{\Phi }\tilde{w}\right\|}^{2}+\lambda_{1}Ttr\left({\tilde{w}}^{T}\Phi \tilde{w}\right)+\lambda_{2}Ttr\left[{\left(d-I\right)}^{T}\left(d-I\right)\right],$$

where $\tilde{Y}={\left[{\tilde{y}}_{1}^{T},{\tilde{y}}_{2}^{T},\ldots ,{\tilde{y}}_{N}^{T}\right]}^{T}$. Let $\frac{\partial J}{\partial d^{T}}=0$ and $\frac{\partial J}{\partial {\tilde{w}}^{T}}=0$, we have

(17) $$R^{T}Q_{1}^{T}Q_{1}Rd+R^{T}Q_{1}^{T}Q_{2}\bar{\Phi }\tilde{w}+\lambda_{2}T\left(d-I\right)=R^{T}Q_{1}^{T}\tilde{Y}$$

(18) $${\bar{\Phi }}^{T}Q_{2}^{T}Q_{1}Rd+{\bar{\Phi }}^{T}Q_{2}^{T}Q_{2}\bar{\Phi }\tilde{w}+\lambda_{1}T\bar{\Phi }\tilde{w}={\bar{\Phi }}^{T}Q_{2}^{T}\tilde{Y}.$$

According to the properties of QR decomposition, we have

(19) $$Q_{1}^{T}Q_{2}=0$$

(20) $$Q_{1}^{T}Q_{1}=I_{D+1}$$

(21) $$Q_{2}^{T}Q_{2}=I_{M-D-1}$$

Insert the above expressions into Equations (17) and (18), then the final solution can be expressed as

(22) $$d={\left(R^{T}R+\lambda_{2}T\;I\right)}^{-1}\left(R^{T}Q_{1}^{T}\tilde{Y}+\lambda_{2}T\;I\right)$$

(23) $$\tilde{w}={\left({\bar{\Phi }}^{T}\bar{\Phi }+\lambda_{1}T\bar{\Phi }\right)}^{-1}{\bar{\Phi }}^{T}Q_{2}^{T}\tilde{Y}.$$

By iteratively determining the correspondences and estimating the spatial transformation, the nonrigid point set registration can be accomplished. It is worth noting that the affine part d and the nonaffine part $\tilde{w}$ are independently calculated, which can avoid mutual inference between d and $\tilde{w}$, and thus, convenient for controlling and analyzing the registration process.

The proposed method is summarized in Algorithm 1.

**Algorithm 1** The proposed method:**Input**: Model point set X and scene point set Y.**Output**: Transformed model point set.**Initialize**: Parameters T, τ, ς, ρ, $\lambda_{1}^{init}$, $\lambda_{2}^{init}$, and K, and probabilities $p_{mn}=1/(MN)$.**Begin**: Construct kernel matrix, and perform the QR decomposition of model point set.
**Repeat:**
Compute the AD and AD-correspondences using Equations (3) and (4), respectively;Compute the RD and RD-correspondences using Equation (5) and Hungarian method, and assign confidence for RD-correspondences using Equations (6)–(8);Compute the collaborative correspondences and corresponding matrix using Equations (9) and (10), respectively;Compute the transformation parameters of affine and nonaffine parts using Equations (22) and (23), respectively;Update T=τT, $\lambda_{1}=\lambda_{1}^{init}T$, and $\lambda_{2}=\lambda_{2}^{init}T$.
**Until**: Achieve the maximum number of iterations;Output the transformed points using Equation (14).

## 4. Annealing Scheme and Convergence Analysis

In the optimization, we adopt the deterministic annealing scheme to control the iterative process. As a heuristic and efficient strategy to escape the poor local minimum points, the deterministic annealing has been broadly employed by many nonrigid point registration methods [16,17,25,29]. The deterministic annealing plays an important role in our method, which is carefully discussed as follows:

(1) Temperature T controls the search range of AD-correspondences, as illustrated by Equations (3) and (4). Temperature T is gradually reduced by a linear annealing schedule $T^{new}=\tau T^{old}$, where τ is the positive annealing rate less than one. At the start, it is hard to determine the accurate one-to-one correspondences so that high temperature T is employed to preserve the corresponding relations with a wide-range. The search range is reduced through gradually reducing temperature T. When T is close to zero, it equivalently uses the hard decisions to determine one-to-one correspondences. 

(2) Temperature T is involved in the process of confidence assignment for each RD-correspondence according to consistent distance, as illustrated by Equations (6)–(8). Under the deterministic annealing strategy, we can gradually exclude RD-correspondences with low consistency, as discussed in Section 3.2. We can also control the roles of RD-correspondences to adjust decay parameter ρ using the deterministic annealing scheme.

(3) Regularization parameters $\lambda_{1}$ and $\lambda_{2}$ are also evolved by following deterministic annealing schedule, $\lambda_{1}=\lambda_{1}^{init}\cdot T$ and $\lambda_{2}=\lambda_{2}^{init}\cdot T$, where $\lambda_{1}^{init}$ and $\lambda_{2}^{init}$ are the two initial values. Through reducing regularization parameters from large to small, the spatial transformation based on TPS is recovered from rigid to nonrigid.

Overall, we adopt a coarse-to-fine and rigid-to-nonrigid strategies to optimize the proposed method based on deterministic annealing techniques. As a dual update process, in each iteration, we can obtain the closed-form solutions of TPS transformation after the correspondences are determined. In next iteration, the newly estimated transformation can be used to help determine the correspondences. Besides, as the temperature decreases, the search range of AD-correspondences is reduced and RD-correspondences with high consistency are emphasized. Thus, we can determine the correspondences more accurately. Next, the newly updated correspondences are utilized to recover the spatial transformation. Given the lower temperature, the spatial transformation can have more freedom on nonrigid terms to recover local nonrigid transformation more accurately. In the end, the correspondences are approximately one-to-one, and the nonrigid transformation is recovered. In summary, the correspondence determination and the spatial transformation estimation run iteratively to gradually converge to a stable local minimum under the deterministic annealing scheme.

## 5. Computational Complexity

In each iteration, the AD-correspondences between two point sets take complexity O(MN). The complexity of preliminary RD-correspondences using Hungarian method is $O\left(M^{3}\right)$. The corresponding confidence assignment needs to find K (K<<M) closest points for each point pairs for which the preliminary RD-correspondence is TRUE. By using sequential search, it costs O(KM) complexity for one point and spends $O\left(2KM^{2}\right)$ for the two point sets. It totally needs $O\left(M^{3}+2KM^{2}\right)$ operations to compute the RD-correspondences. Solving the nonrigid transformation needs complexity $O\left(M^{3}\right)$. Overall, the complexity of the proposed method takes $O\left(M^{3}\right)$.

## 6. Results

Our method is implemented in MATLAB, and the experimental environment is an Intel Core i7-7700 CPU and 32GB RAM. We choose the methods which have publicly available codes that are provided by the authors, and keep their default parameter settings, including TPS-RPM [17], CPD [19], GLMDTPS [28], MR-RPM [33], and GLTP [36]. The root-mean-square error (RMSE) is used as the registration error, which is denoted as follows

(24) $$RMSE=\sqrt{\frac{1}{M}\sum_{m=1}^{M}{\left\|f\left(x_{m}\right)-{\overset{⌢}{y}}_{m}\right\|}^{2}},$$

where ${\overset{⌢}{y}}_{m}$ is the ground truth corresponding point of $x_{m}$.

*Parameter settings*: The $T_{init}$, $T_{final}$, and annealing rate τ are used to control the deterministic annealing process. As the initial temperature, $T_{init}$ is high. The stopping temperature $T_{final}$ is low. However, they are not too high or too low, otherwise, they would spend much more computation to accomplish the registration. We experimentally recommend to set $T_{init}\in \left[0.1,0.5\right]$, $T_{final}\in \left[10^{-3},10^{-5}\right]$, and τ∈[0.90,0.98]. In our paper, we fix $T_{init}=0.2$, $T_{final}=T_{init}/1500$, and τ=0.93. We keep these settings in the following experiments. In each temperature, the method runs three iterations. In order to save computation, we only calculate RD-correspondences once in each temperature.

Parameters that are related to RD-correspondences include parameters of SC, and parameters ρ and K. We preserve the default settings of SC in [11]. Parameter ρ controls the roles of RD-correspondences in collaborative correspondences. The proposed method can perform well for ρ=0 in the outlier-free scenarios. When there are outliers in data, we set ρ=N/(4M). Parameter K is the number of neighboring points that are employed to evaluate the corresponding consistency of RD-correspondences. Large K can underline the RD-correspondences with the high corresponding consistency, in order to obtain more accurate RD-correspondences. However, if K is too large, it would be too strict to preserve sufficient RD-correspondences. Simultaneously, computational cost will increase. In our paper, by taking a balance of these factors, the number of neighboring points is set as K=3 and K=7 when M=N and M≠N, respectively.

Parameter ς is used to handle outliers. Regularization parameters $\lambda_{1}$ and $\lambda_{2}$ are employed to trade off the flexibility and smoothness of the spatial transformation. As discussed in [17], the $\lambda_{2}^{init}$ is set as much smaller than $\lambda_{1}^{init}$ for providing more freedom for the affine transformation. In our paper, we fix ς=0.5 and $\lambda_{2}^{init}=0.01\lambda_{1}^{init}$. We set initial value $\lambda_{1}^{init}$ as 0.5 and $\lambda_{2}^{init}=0.01\lambda_{1}^{init}=0.005$.

### 6.1. Results on Fish Dataset

We test our algorithm on fish data in [17,27]. Each fish model point set contains 98 points. There are five kinds of degradations, including nonrigid deformation, noise, outlier, rotation, and occlusion. For each degradation, there are five levels. For each level, there are 100 samples. We give the qualitative and quantitative results. In quantitative results, the registration results of all the 100 samples for each level are used to evaluate the performance of the nonrigid point set registration algorithms. Notably, as demonstrated in [28], there are two cases of occlusion: missing points in one side and both sides. Our method can achieve good performance in first case. In the second case, our method enforces each model point to search its corresponding point in the scene point set, while the corresponding point might not exist. Thus, our method cannot handle the second case well. In the experiments of occlusion, we present the performance of the methods with missing points in single side. 

**Figure 1:**
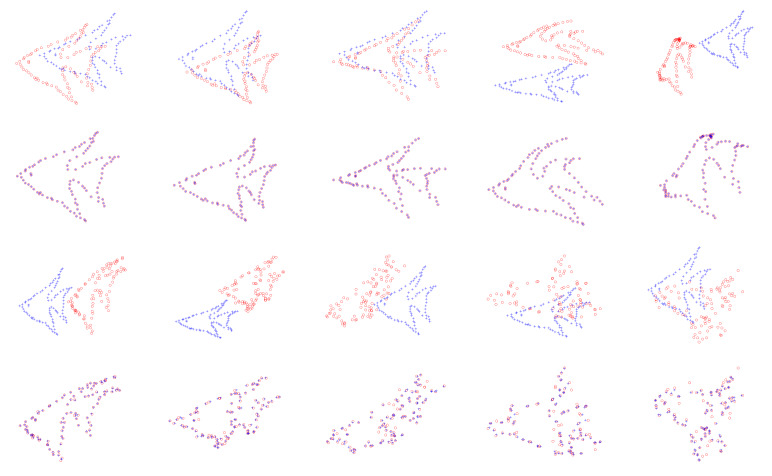

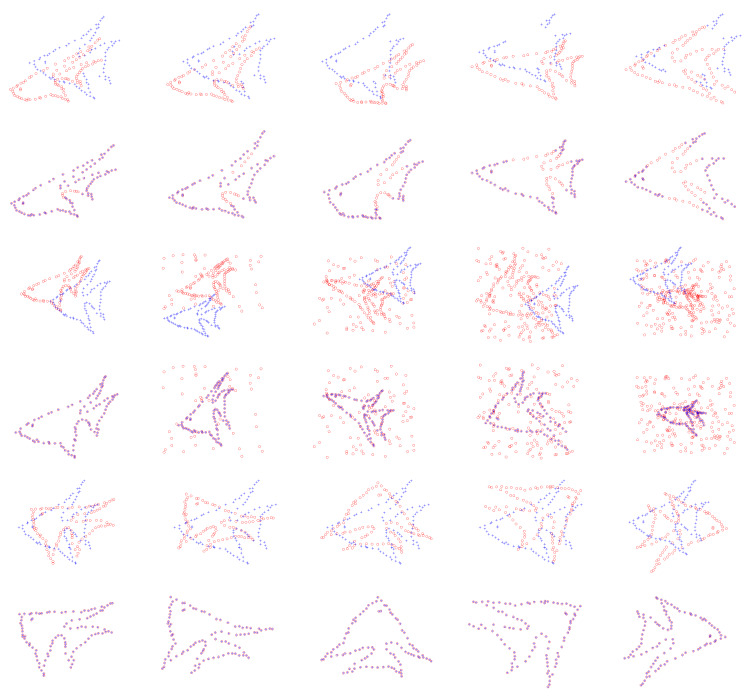
Registration results on fish dataset. From top to bottom: deformation, noise, occlusion, outliers, and rotation in every two rows; the upper row shows the data and the lower row shows the registration results. From left to right, the degradation level becomes larger. The model points and the scene points are marked by “+” and “o,” respectively.

Figure 1 shows qualitative results on some examples of fish dataset. The model points are represented by “+,” and the scene points are denoted by “o.” The results are arranged by every two rows, the upper row shows the data and the lower gives the registration results. From top to bottom, there are different degradations including deformation, noise, occlusion, outliers, and rotation. From left to right, the degradation level gradually increases. The proposed method is accurate and robust in the degradations of deformation, low noise, occlusion (single side), outliers, and rotation. When the noise level is high, the structures are severely damaged. The proposed method can roughly recover the spatial transformation.

**Figure 2:**
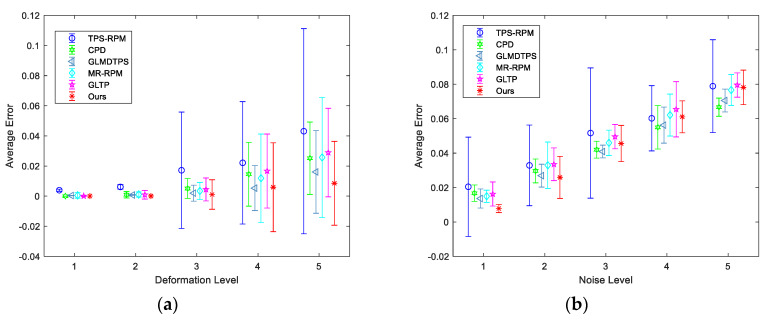

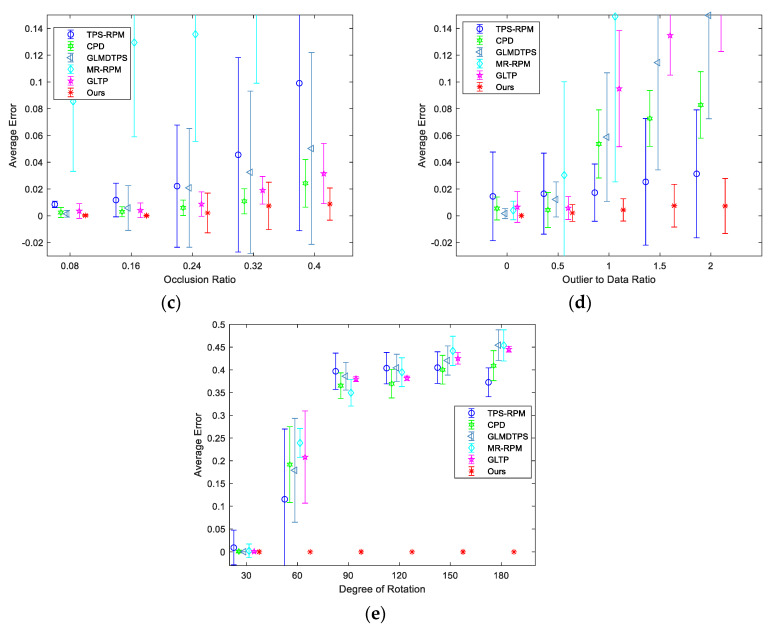
Quantitative comparisons of TPS-RPM, CPD, GLMDTPS, MR-RPM, and GLTP, and the proposed algorithm on fish dataset. (**a**–**e**) Registration results under degradations of deformation, noise, occlusion, outliers, and rotation, respectively. The error bars indicate the mean and standard deviation of registration errors over 100 trials.

Figure 2 reports the quantitative results of seven state-of-the-art methods and ours. In the test of deformation, as illustrated in Figure 2a, when the deformation is not large, all the methods can obtain good results in the registration accuracy. Notably, our method can still keep perfect performance as the deformation becomes large. Figure 2b shows the results of the registration methods under the degradation of noise. Our method can get the best results in low noise level. When the noise level is high, the structures of the point sets are not well preserved so that the performance declines. Figure 2c shows the registration results under the degradation of occlusion. The ratios of missing points are from 0.08 to 0.40 with an interval of 0.08. We can see that the proposed method can keep better performance than other methods when some points are missing. Figure 2d presents the results of the registration methods under the degradation of outliers. CPD and GLMDTPS can perform well when the number of outliers is small, but cannot handle large number of outliers well. We observe that our method shows outstanding performance when dealing with outliers. Figure 2e shows the registration results in rotation. We rotate the data from 30° to 180° with an interval of 30°. As illustrated in [27], by using the direction from a point to the mass center of a point set as the positive X-axis for the local coordinate system, it can get rotation invariant shape context (RISC). We adopt RISC to correct the rotation between two point sets at the start. This can make our method be invariant to the rotation.

### 6.2. Results on Chinese Character Dataset

Chinese characters are pictographic and have abundant and meaningful structures. The Chinese character dataset can be obtained from https://github.com/xdregis/complicated_chinese_characters. To evaluate the performance of the registration methods, we choose five Chinese characters with complicated structures, namely, “cake,” “dim,” “math,” “micro,” and “tree,” with 148, 153, 177, 189, and 149 points, respectively. The Chinese characters are handwritten, and the feature points are manually annotated as the model point set. The nonrigid deformation versions of the model point set are made by the techniques in [28] as scene point sets. There are five levels for nonrigid deformation. For noise degradation, we add Gaussian white noise to the data with five levels. The mean of the noise is zero, and the standard deviation is changed from 0.06 to 0.18 with an interval of 0.03. For occlusion degradation, the ratios of missing points are from 0.08 to 0.40 with an interval of 0.08. For outlier degradation, we add random distributed outliers to data. The ratios of outliers to data are from 0.2 to 1.0 with an interval of 0.2. For rotation degradation, we rotate the data from 30° to 180° with an interval of 30°. For each level, each character has 100 samples. Therefore, five characters have 500 examples.

**Figure 3:**
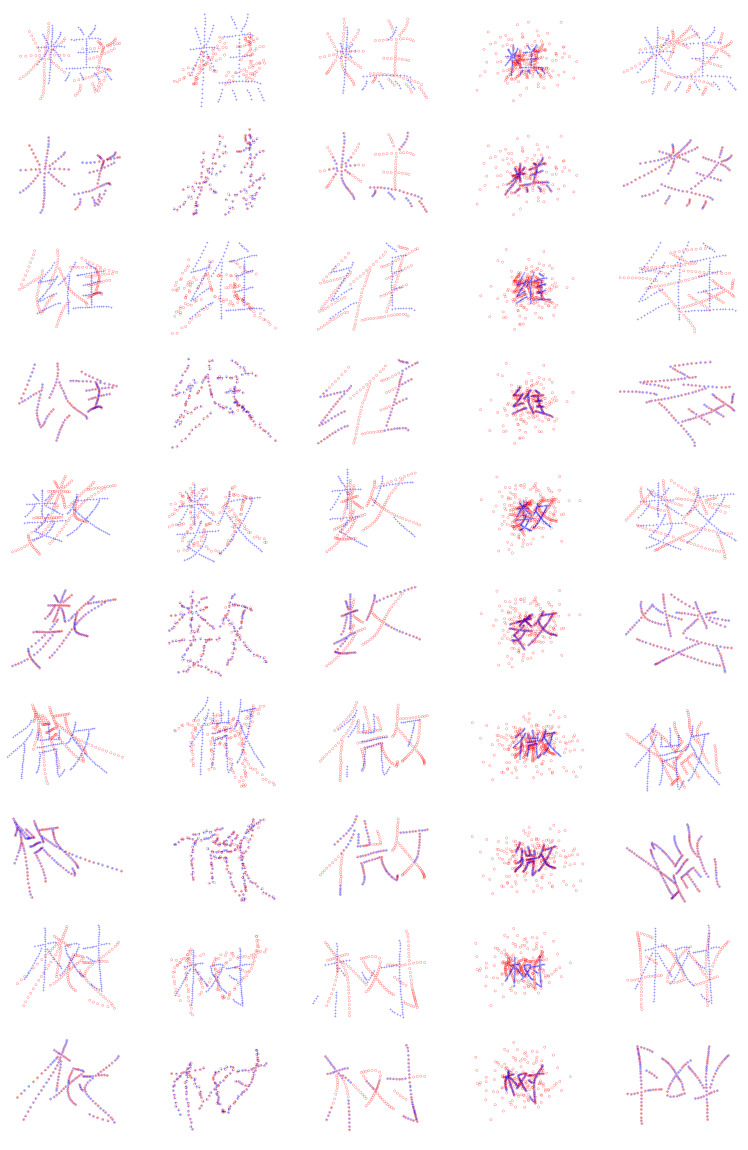
Registration results on Chinese character dataset. From top to bottom: “cake,” “dim,” “math,” “micro,” and “tree” in every two rows; the upper row shows the data and the lower row shows the registration results. From left to right: deformation, noise, occlusion, outliers, and rotation. The model points and the scene points are marked by “+” and “o,” respectively.

**Figure 4:**
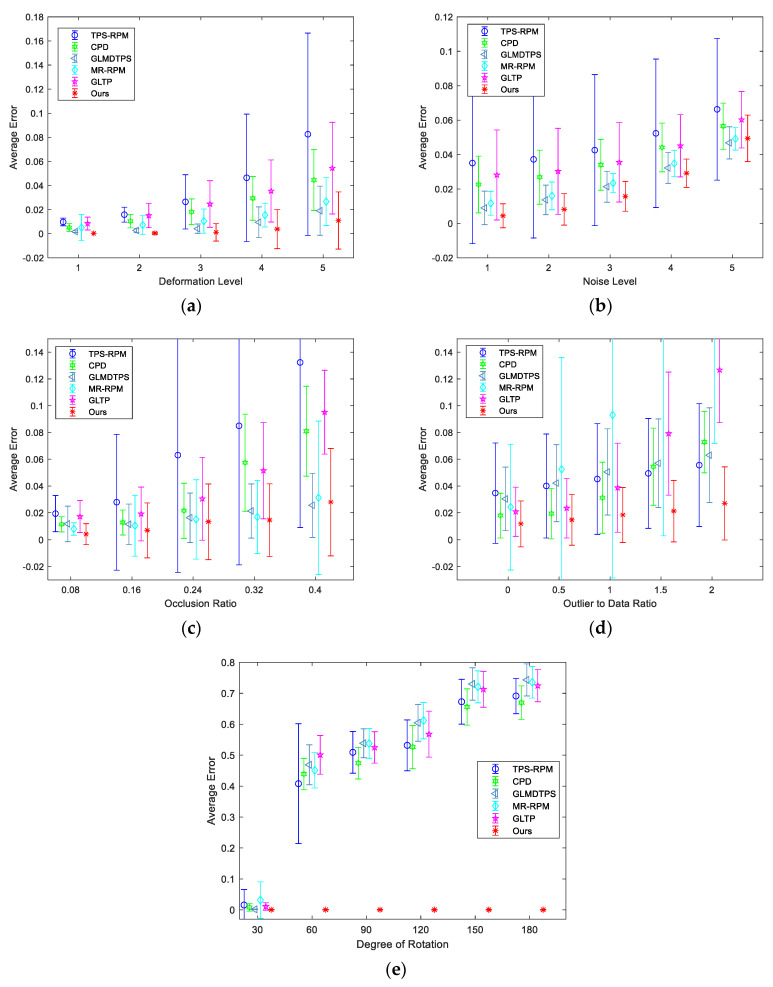
Quantitative comparisons of TPS-RPM, CPD, GLMDTPS, MR-RPM, and GLTP, and the proposed algorithm on Chinese dataset. (**a**–**e**) Registration results under degradations of deformation, noise, occlusion, outliers, and rotation, respectively. The error bars indicate the mean and standard deviation of registration errors over 500 trials.

Figure 3 shows the qualitative results of the proposed method on some examples of the five Chinese characters on the degradations. We can see that the structures of Chinese characters are complicated because they are formed by several relatively independent parts. The experimental results illustrate that the proposed method can accurately align model point sets to scene point sets. Figure 4 shows the quantitative results on the Chinese characters of the seven state-of-the-art methods and ours. As shown in Figure 4a, our method is most accurate in deformation. In Figure 4b, our method shows the best performance from levels 1–4 in noise. As illustrated by Figure 4c, in occlusion (single side), GLMDTPS, MR-RPM, and our methods are accurate and robust in five levels. In Figure 4d, our method achieves the outstanding performance in the degradation of outliers. Figure 4e shows the registration results in rotation. Our method is invariant to the rotation.

### 6.3. Results on IMM Face Dataset

**Figure 5:**
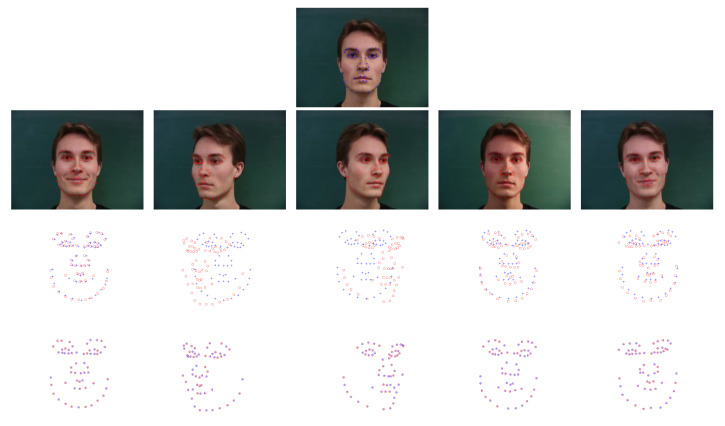
Registration results on some examples on the IMM face dataset. The first row is the original image of view 1, and the annotated landmarks are employed as model point set. The second row is the original images from view 2 to view 6, and the annotated landmarks are employed to the scene point sets. The third and fourth rows are the point sets to be registered and the registration results, respectively. The model points are marked by “+,” and the scene points are marked by “o.”

**Figure 6:**
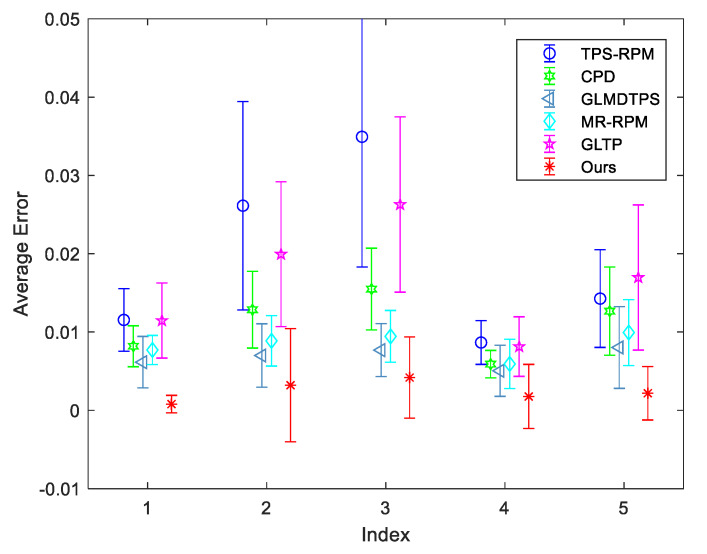
Quantitative comparisons of TPS-RPM, CPD, GLMDTPS, MR-RPM, and GLTP and the proposed method on IMM face dataset. From Group 1 to Group 5, they represent the registration results between view 1 and view 2 to view 6, respectively. The error bars indicate the mean and standard deviation of registration errors over 40 trials.

IMM face dataset consists of 240 annotated monocular images of 40 different human faces. Each face is annotated by 58 feature points. For each human, there are six views with different expressions and angles. We take the first view as the model point set, and the others are used as the scene point sets. Thus, five groups are defined as Group 1 to Group 5. Each group has 40 pairs of point sets to be matched. The qualitative results on one of the persons are shown in Figure 5. The first row is the face of view 1. This face is employed as the model face. The second row is the faces from view 2 to view 6. These faces are employed as the scene faces. We can see that the facial expressions and angles are significantly changed from view 1 to view 3. The third row shows the point sets to be matched. The fourth row shows the registration results. The proposed method can accurately align the point sets even though the facial expressions and angles are very different. The quantitative results of five groups are provided in Figure 6. We can see that the proposed method achieves the best performance.

### 6.4. Results on IMM Hand Dataset

**Figure 7:**
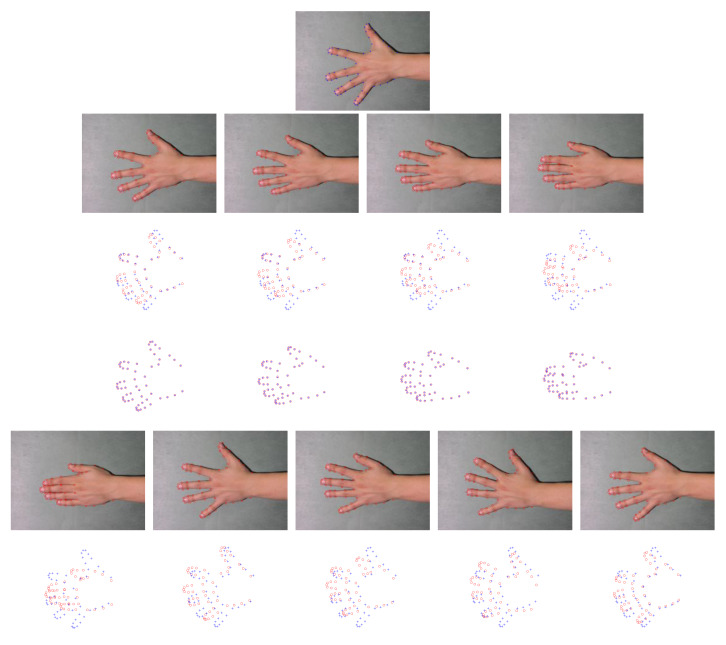

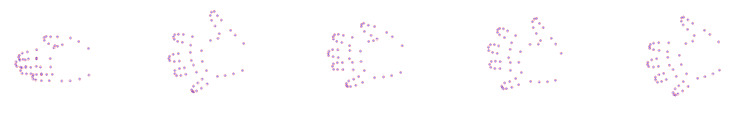
Registration results on some examples on the IMM hands dataset of person 2. The first row is the original image of gesture 1, and the annotated landmarks are employed as model point set. The second row and fifth row are the original images from gesture 2 to gesture 5 and gesture 6 to 10, respectively. Their annotated landmarks are employed to the scene point sets. The third and sixth rows are the point sets to be registered and the fourth and seventh rows are the registration results. The model points are marked by “+,” and the scene points are marked by “o.”

**Figure 8:**
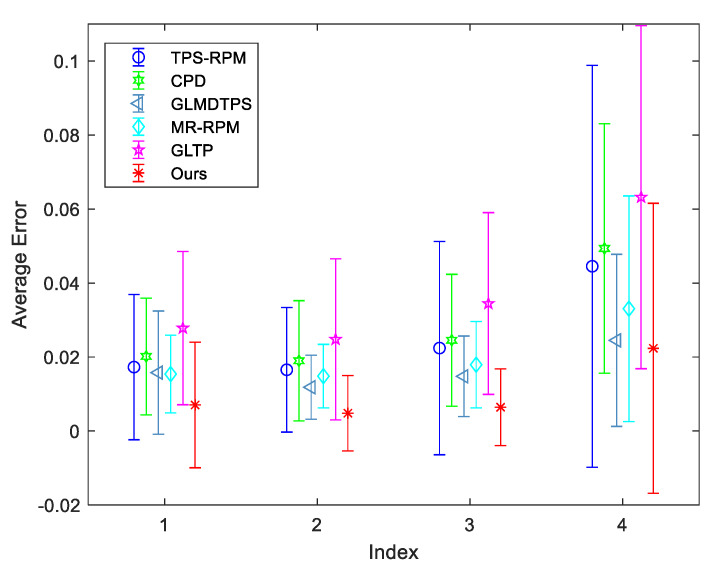
Quantitative comparisons of TPS-RPM, CPD, GLMDTPS, MR-RPM, and GLTP, and the proposed method on IMM hands dataset. From index 1 to 4, they represent the registration results from Group 1 to Group 4, respectively. The error bars indicate the mean and standard deviation of registration errors over 90 trials.

**Figure 9:**
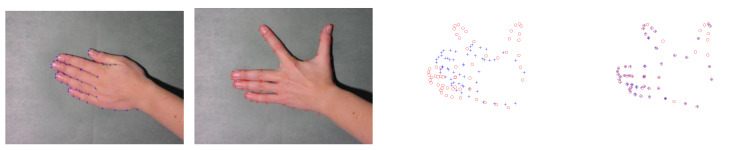
A failure case in Group 4 of IMM hands dataset. From left to right: the model image, the scene image, the original point sets, and the registration results. The model point sets are marked by “+,” and the scene point sets are marked by “o.”

IMM hands dataset consists of 40 annotated monocular images with resolution pixels 800×600 from four different human hands, and each person provides 10 images with different hand gestures. In each group, each gesture is employed as the model data in turn. The other nine gestures are employed as the scene data. Thus, there are 90 different combinations for each group. The qualitative results of gestures from one person are shown in Figure 7. We take the first gesture as the model point set, and the other nine gestures are used as the scene point sets. Although the hand gestures have significant changes, the proposed method can accurately recover the spatial transformation. Figure 8 presents the quantitative results. As illustrated by Figure 8, the proposed method can get the highest accuracy from Group 1 to Group 4. In the Group 4, our method is still most accurate, but the accuracy is lower than previous groups, and the standard deviation is large. Although our method can obtain good registration results in most cases, several examples in Group 4 are hard to be well matched by ours. We give a failure case in Figure 9. As shown in the first and second subfigures in Figure 9, we can see that the adjacent fingers of the model gestures are very close, and the feature points that represent these fingers approximatively line up on the boarders. Although these points belong to different fingers, their structural features are similar so that they are difficult to be correctly separated when they are employed as the model points. Besides, the spatial transformation between two gestures is large. This further increases the difficulty to accurately match the point sets. Our method is not very well to handle these cases. In Group 4, the hand gestures are more than others, and thus, this results in performance degradation.

Finally, in Table 1, we give the comparisons of runtime of point set registration methods on fish (98 points), cake (138 points), dim (153 points), math (177 points), micro (189 points), tree (149 points), IMM face (58 points), and IMM hands (58 points). CPD, GLMDTPS, and GLTP are implemented by C and MATLAB. Although the computation complexity is in same level, they run much faster than the methods that are only implemented by MATLAB, including TPS-RPM, MR-RPM, and ours. TPS-RPM is the most relevant method to ours. Both TPS-RPM and our methods employ TPS as the transform function and adopt the deterministic annealing as the optimization strategy. TPS-RPM only needs to calculate AD-correspondences. In order to get more accurate correspondences, our method has to consume more time to calculate RD-correspondences besides AD-correspondences. In summary, our method can efficiently improve the registration accuracy, but the computation cost increases.

## 7. Conclusions

In this paper, we have presented a robust and accurate method and its applications for nonrigid point set registration. The main idea of our method is to find an efficient way to combine AD-correspondences and RD-correspondences to determine the corresponding relationship between the point sets to be matched. AD-correspondences and RD-correspondences are derived from the structures with different attributes. They are complementary and are able to improve the utilization efficiency of the abundant structural information within the point sets. Moreover, TPS is adopted as the transformation function. At each iteration step, the closed-form solutions of the affine and nonaffine parts have been used, which makes it be convenient to analyze and control the registration process. Experiments illustrate that the proposed method can achieve good performance on both synthetic and real data.

Although the proposed method can achieve good performance in most scenarios of nonrigid point set registration, the proposed method cannot handle the degradations well when the miss points are on both sides. Our method has relatively heavy computation when there are a large number of points, such as the 3D point clouds that are obtained by RGB-D cameras, laser scanners, and lidar, which usually contain thousands of points. In the future, we will focus on finding efficient way to reduce the computational complexity.

## Figures and Tables

**Table 1:** Runtime(s) of point set registration methods on different datasets.

	Fish	Cake	Dim	Math	Micro	Tree	IMM Face	IMM Hands	Mean Runtime
TPS-RPM	0.841	1.537	1.836	2.331	2.262	1.737	0.380	0.366	1.411
CPD	0.049	0.095	0.115	0.130	0.162	0.101	0.020	0.022	0.087
GLMDTPS	0.092	0.122	0.126	0.143	0.155	0.123	0.065	0.063	0.111
MR-RPM	2.082	1.669	1.595	2.105	2.406	1.930	0.176	0.196	1.520
GLTP	0.075	0.133	0.177	0.216	0.248	0.162	0.023	0.023	0.132
Ours	1.703	3.959	3.690	5.037	5.679	3.534	0.699	0.695	3.158
